# Soft tissue-based surgical techniques for treatment of posterior shoulder instability

**DOI:** 10.1007/s11678-017-0413-5

**Published:** 2017-05-24

**Authors:** Alessandro Castagna, Marco Conti, Raffaele Garofalo

**Affiliations:** Shoulder and Elbow Unit, IRCCS Humanitas Institute, Via Manzoni 56, 20089 Rozzano (Milan), Italy

**Keywords:** Soft tissue injuries, Shoulder dislocation, Joint instability, Glenohumeral joint, Arthroscopy, Weichteilverletzungen, Schulterluxation, Gelenkinstabilität, Glenohumeralgelenk, Arthroskopie

## Abstract

Posterior shoulder instability is a rare clinical condition that encompasses different degrees of severity including various possible pathologies involving the labrum, capsule, bony lesions, and even locked posterior dislocation. When focusing on soft tissue involvement, the diagnosis of posterior instability may be difficult to make because frequently patients report vague symptoms not associated with a clear history of traumatic shoulder dislocation. Pathological soft tissue conditions associated with posterior instability in most cases are related to posterior labral tear and/or posterior capsular detensioning/tear. The diagnosis can be facilitated by physical examination using specific clinical tests (i. e., jerk test, Kim test, and reinterpreted O’Brien test) together with appropriate imaging studies (i. e., magnetic resonance arthrography). Arthroscopy may help in a complete evaluation of the joint and allows for the treatment of soft tissue lesions in posterior instability. Caution is warranted in the case of concomitant posterior glenoid chondral defect as a potential cause of poor outcome after soft tissue repair in posterior instability.

## Zusammenfassung

Die posteriore Schulterinstabilität ist eine seltene klinische Manifestation, die unterschiedliche Schweregrade einschließlich verschiedener möglicher pathologischer Veränderungen an Labrum oder Kapsel, knöcherne Läsionen und selbst die verhakte posteriore Schulterluxation umfasst. Konzentriert man sich auf die Beteiligung der Weichteile, kann die Diagnose der hinteren Instabilität schwierig zu stellen sein, da die Patienten häufig vage Symptome angeben, die nicht mit der klaren Anamnese einer traumatischen Schulterluxation einhergehen. Pathologische Veränderungen der Weichteile mit posteriorer Instabilität gehen zumeist mit einem hinteren Labrumriss und/oder einer Entspannung/einem Riss im Bereich der hinteren Kapsel einher. Die Diagnose kann durch die körperliche Untersuchung mit spezifischen klinischen Tests (d. h. Jerk-Test, Kim-Test und neu interpretierter O’Brien-Test) zusammen mit entsprechender Bildgebung (d. h. Magnetresonanzarthrographie) vereinfacht werden. Die Arthroskopie kann zur vollständigen Beurteilung des Gelenks beitragen und ermöglicht die Behandlung von Weichgewebsläsionen bei posteriorer Instabilität. Vorsicht ist im Fall eines begleitenden hinteren glenoidalen Knorpeldefekts als mögliche Ursache eines ungünstigen Ergebnisses nach Weichteilrekonstruktion bei posteriorer Instabilität angebracht.

Posterior shoulder instability is a relatively rare condition representing about 10% of all cases of shoulder instability [13].

Different conditions inducing posterior instability have been described including acute traumatic conditions, chronic locked conditions, and painful unstable shoulder. A posterior recurrent shoulder instability is rarely observed. In this review we focus on the treatment of symptomatic posterior unstable glenohumeral joint without bone component involvement and/or associated pathologies.

## Classification

Posterior instability includes a wide spectrum of pathological conditions, which are sometimes difficult to classify homogeneously.

From an etiological point of view, the posterior dislocation is typically differentiated into traumatic nonlocked dislocation, locked dislocation, and chronic instability. Locked dislocation can be further differentiated into acute or chronic depending on the time elapsed from the trauma and diagnosis. Normally, after 6 weeks a locked posterior dislocation should be considered chronic.

### Traumatic nonlocked posterior dislocation

An acute traumatic complete posterior dislocation is seldom reported and therefore the incidence of this kind of instability is poorly studied. This dislocation may occur in patients with seizures because of uncoordinated muscle contraction, as a consequence of a posteriorly directed blow (motor crash or sports contact), or after a violent fall, even in a domestic environment. In these situations, patients report pain with active motion and particularly limited external rotation of the arm.

### Locked posterior dislocation

In the case of locked posterior dislocation, no external rotation is possible even with passive motion. In these cases, the situation may mimic a frozen shoulder on examination, especially in cases with a chronic (neglected) unreduced dislocation. Appropriate imaging to detect a complete posterior dislocation must be performed, and if the suspected diagnosis is confirmed an early closed reduction is required followed by immobilization in external rotation. In the case of chronic unreduced dislocation, an open reduction may be necessary in association with other treatments according to the articular damage.

### Chronic instability

A more chronic situation with recurrent posterior instability, mostly subluxation, is even more challenging to identify because the patient’s symptoms are vague and clinical examination is not straightforward. In fact, patients with posterior shoulder instability do not report a history or symptoms that can be directly linked to a particular event or injury. Posterior instability often overlaps with laxity in its clinical presentation, thus contributing to a misleading scenario. In this particular group of patients, some repetitive activities such as cross-arm abduction, bench press, or overhead throwing can result in microtraumatic events causing a prevalent posterior shoulder instability in a setting of laxity or multidirectional instability.

Many of these patients report symptoms such as fatigue, pain, and eventually loss of strength in elevation. Rarely do patients report clear posterior shoulder pain associated with the feeling of a dead arm.

### Classification groups

For the aforementioned reasons, the classification of posterior instability may be complex or confusing. Nevertheless, an attempt was made to classify symptomatic posterior instability into four groups [23]:

- Group A: predominantly posterior multidirectional instability, termed “PPM”
- Group B: recurrent posterior subluxation, termed “RPS”
- Group C: traumatic unidirectional posterior instability
- Group D: traumatic posterior instability with additional anterior lesions (“bidirectional”)

Patient age is considered another important factor for possible associated lesions. Often, younger patients do not have associated lesions, unlike older patients, and consequently the treatment algorithm for these patients is different. Furthermore, a chronic neglected dislocation in an older patient may have different surgical solutions independently of the associated lesions.

Traditionally, treatment for posterior shoulder instability consisted of conservative management. Surgery is required in cases of persistent shoulder pain in chronic instability, in patients with a locked neglected chronic dislocation, or in patients with associated lesions developing a dysfunctional shoulder.

Once the diagnosis of posterior instability is suspected or confirmed in patients with chronic painful shoulder, arthroscopy is considered the gold standard for the detection and treatment of lesions that typically develop with posterior instability, which may be hidden or subtle. Mixed results have been reported in the series published to date [1, 16, 23].

## Pathophysiology

The glenohumeral joint is often described as a ball and socket joint. However, the glenoid socket is very shallow and therefore in the absence of bone defects (humeral or glenoid or combined) the stability of this joint relies on the labrum, the ligaments, the musculature of the shoulder girdle, and on neuromuscular control.

### Posterior inferior glenohumeral ligament

The posteroinferior capsule is not as robust as the anterior capsule and the posterior inferior glenohumeral ligament (PIGHL) is thinner than the anterior counterpart [4]. The PIGHL is the most important stabilizing ligament particularly in positions of flexion, adduction, and internal rotation of the arm. The subscapularis muscle is considered to be the most important dynamic posterior stabilizer, whereas the role of the rotator interval as a posterior stabilizer is controversial [15, 24].

### Chondrolabral complex

The cartilage and labrum are important to deepen the glenoid socket and improve the concavity-compression mechanism of stability. Cadaveric studies outlined the importance of the chondrolabral complex in stability, and these findings were confirmed by clinical investigations. Some authors showed that the loss of chondrolabral containment posteroinferiorly is a consistent finding in posteroinferior instability and is principally due to the loss of posterior labral height and detensioning of the ligament complex [5, 17].

### Labral injury

Labral injury from repetitive posterior loading or instability can range from a posterior labral tear to an incomplete, concealed avulsion of the posteroinferior labrum (also known as “Kim lesion”) to a reverse Bankart lesion. Capsular tearing, posterior labrocapsular periosteal sleeve avulsion, or humeral avulsion of the posteroinferior glenohumeral ligament (PHAGL) also can occur and contribute to the recurrence of posterior instability [6, 27, 29].

It has been reported that a redundant or patulous capsule is the main cause of posterior instability, with a reported incidence of labral lesions at the time of surgery varying between 10 and 100% [1, 16, 20]. However, while many of these may be obvious on arthroscopy, nearly 40% are discrete lesions [20].

Furthermore, posterior instability can be related to a dysplastic condition such as excessive glenoid retroversion or humeral head torsion. Some forms of instability can be acquired and related to (a) scapulothoracic dysfunction based on overuse or failure of a muscle–tendon unit, (b) a neurological deficit, or (c) a bony or posterior soft tissue insufficiency.

In summary, the occurrence or recurrence of traumatic posterior dislocation is very rare. Most posterior shoulder instabilities can be classified as an acute traumatic event or as the result of repetitive microtraumatic events often associated with a laxity (multidirectional instability with a posterior prevalence). In these situations there is a repetitive microtrauma in the form of rim loading that can create a deformation and elongation of the capsule and labral tear, thus compromising the concavity mechanism of stability [17, 19, 23].

## Imaging

### Radiography

Posterior dislocation may be missed initially on posteroanterior radiographs in 50% of cases, as the humeral head appears to be almost normally aligned with the glenoid. However, different signs, such as the trough line sign or loss of the normal half-moon overlap sign, have been described on standard radiographs as being suggestive of a posterior dislocation. An axillary view is the preferred view for diagnosis, while a Velpeau or Wallace view is an alternative. A scapular Y view has been shown to be unreliable for diagnosing posterior shoulder dislocations [14].

### Magnetic resonance arthrography

In the case of suspected posterior instability, the best imaging for evaluating the labrum and capsule is magnetic resonance arthrography (MRA). A computed tomography (CT) scan can be useful when a better evaluation of the bone deformity (glenoid dysplasia or retroversion), or of the size of the humeral head defect in cases of locked posterior dislocation, is required. With static MRA one is able to check glenoid labrum tears, labral height, and labral contour.

A redundant or patulous posterior and inferior capsule can be assessed on MRA. However, when assessing capsular redundancy it is very important to take into consideration the amount of contrast medium injected, the gravity-dependent filling, and the position of the arm. Classically, MRA is performed with the patients in supine position and, because of gravity, contrast medium tends to accumulate in the posterior capsule; as a consequence, there may be some false-positive findings. MRA can also be useful for identifying PHAGL lesions that, although rarely, may be the cause of a posterior instability [6].

## History and clinical examination

As mentioned, the clinical symptoms in posterior instability range from posterior pain, fatigue, weakness, and, rarely, instability and may be associated with mechanical symptoms such as clicking or catching. Symptoms intensify with the arm in 90° forward flexion, adduction, and internal rotation. The clinical tests used in examinations of posterior instability have evolved. The posterior apprehension test and the load and shift test are no longer believed to be useful tests for this condition [16].

### Jerk test

The jerk test (posterior stress test) has been considered to be highly sensitive for posterior instability. This test is performed by stabilizing the scapula with one hand, while the other hand holds the elbow with the arm in 90° of abduction and internal rotation. A firm axial compression force is applied on the glenohumeral joint. The arm is horizontally adducted while maintaining the firm axial load.

### Kim test

A further test for posterior instability is the so-called Kim test, which is a modification of the jerk test [16]. The test is performed with the patient in a sitting position and their arm in 90° of abduction. The examiner holds the elbow and lateral aspect of the proximal arm and applies a strong axial load in line with the scapula. While maintaining the axial load, the arm of the patient is elevated 45° diagonally upward and a posterior force is applied to the proximal arm. The test is positive if the patient complains of pain during this maneuver.

These two clinical tests are very important and are the hallmark for deciding between operative and nonoperative treatment. A painful jerk test or Kim test is suggestive of a labral lesion and, even if this is not confirmed by an MRI examination, arthroscopic treatment with capsuloplasty is recommended. Patients with a painful jerk or Kim test have an approximately 85% chance of not improving with rehabilitation and are candidates for arthroscopic capsuloplasty. Sometimes patients with a posterior subtle instability will complain only of a painful shoulder and the aforementioned test results may be negative.

### O’Brien test

Recently, Owen et al. noted that in patients with a posterior labral tear the O’Brien test can be positive. In contrast to SLAP tear, in these cases the positivity of the test consists not in the pain, but in the discomfort and secondary weakness. According to the study, the O’Brien test is highly predictive (90%) of posterior labral tear [22].

## Conservative treatment

### Physical therapy

If posterior instability is suspected after a clinical examination, physical therapy should be offered to the patient. Physical therapy is focused on strengthening the rotator cuff, posterior deltoid, and the scapular stabilizers through resisted external rotation exercises [21]. This program can reduce instability recurrence and pain and increase function, mainly in those with atraumatic posterior instability [21]. The aim is to improve the neuromuscular control of the shoulder joint. During conservative treatment, patients should modify their activity levels to prevent further injury Conservative treatment has been reported to be less successful for instability that has occurred after a single injury to the shoulder [11] and in patients with a painful jerk or Kim test [16]. Patients who have undergone at least 6 months of conservative treatment and still experience symptoms are candidates for surgical management [12].

## Arthroscopic surgery

### Patient positioning

We use a lateral decubitus positioning of the patient with a beanbag to avoid pressure injury. Combined anesthesia with interscalene block and general anesthesia is suitable to obtain complete muscle relaxation. The affected arm is placed in a balanced arm traction in 45° of abduction and 15° of forward flexion; 4–5 kg is used for the arm traction.

### Surgical technique

**Fig. 1:**
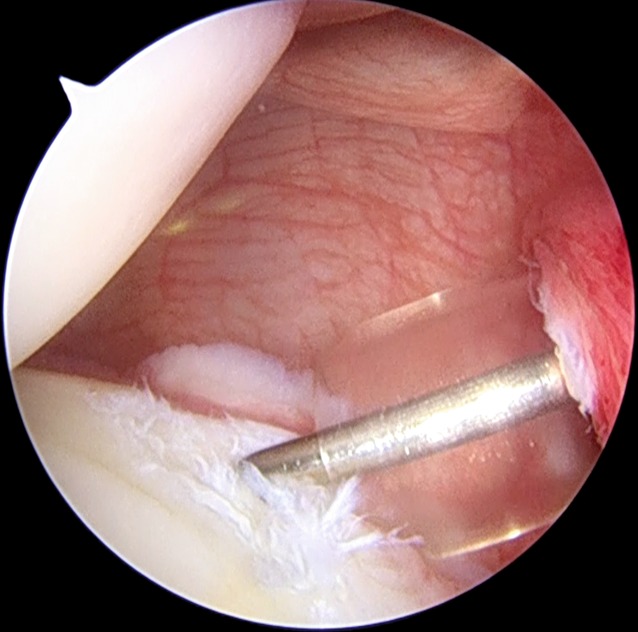
Arthroscopic intraoperative view of a right shoulder. Patient is in lateral decubitus position, the scope is in the anterosuperior portal. A posterior labral tear is shown

A standard posterior portal is made at the beginning. This portal is made in the “soft spot” approximately 2 cm medial and 2 cm inferior to the posterolateral corner of the acromion. It should allow for anchor placement with 45° of angulation relative to the glenoid surface. An anterior portal is made with an in–out or out–in technique according to the surgeon’s preference, and should be made in the rotator cuff interval just inferior to the biceps tendon. The anterior portal is used to introduce the scope and the posterior portal is the working portal. We put a cannula through the portals. Observing from the anterior–superior portal, the labral and capsular pathology is identified (Fig. 1). In the case of labral pathology, the labrum is elevated from the posterior glenoid margin using a periosteal elevator.

**Fig. 2:**
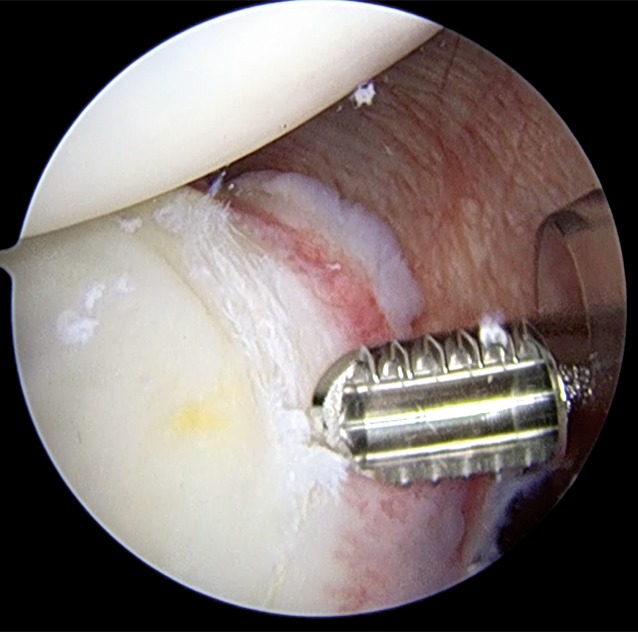
A shaver is used to abrade the labral tear

**Fig. 3:**
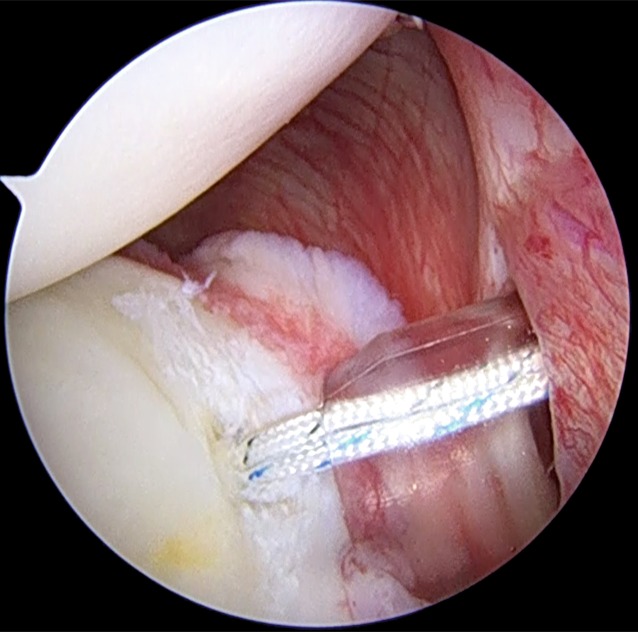
Placement of a double-loaded suture anchor along the posterior glenoid surface. The anchor is inserted through the posterior cannula

The rim and neck of the glenoid are debrided with a shaver (Fig. 2). In patients in whom the position of the posterior portal is not optimal for working and for achieving correct anchor trajectory, particularly for the most inferior anchor, an accessory posterosuperior portal can be used [8]. The anchor is placed along the posterior glenoid margin, starting inferiorly and progressing superiorly, as needed. Different anchors may be used according to the surgeon’s choice, but the general principles of the surgical technique should be respected (Fig. 3). When more than one anchor is used, they should be spaced 3–5 mm apart to avoid fragmentation of the posterior glenoid bone.

**Fig. 4:**
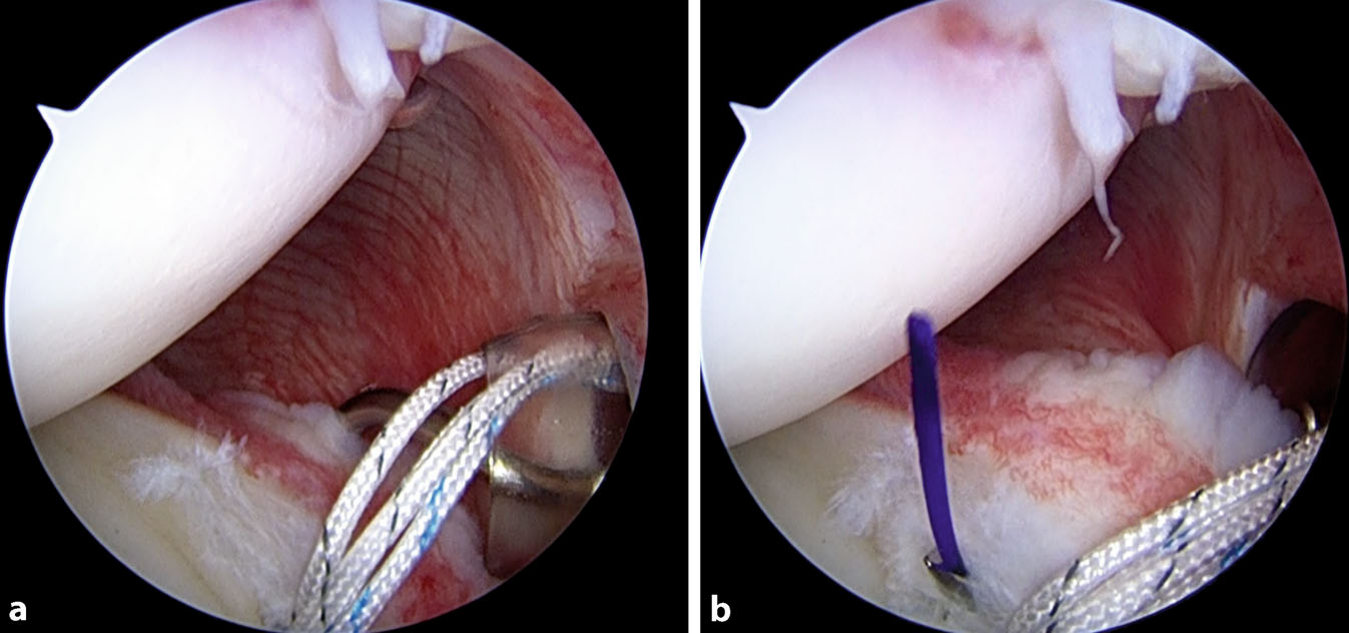
Arthroscopic view showing the use of a suture hook. **a** The 45°-angled hook is inserted through the posterior cannula and keeps the capsular tissue distal to the anchor. **b** The exit of the hook is at the level of the labral tear, near the anchor, for a south-to-north capsular shift

In the case of a labral tear, once that anchor is inserted, we use a 45° spectrum hook system (ConMed Linvatec, Largo, Fl, USA) with polydioxanone (PDS; Ethicon Inc., Somerville, N.J.) #0 as a shuttle suture to penetrate the capsular tissue slightly inferior and lateral to the labrum and anchor so as to perform an inferior-to-superior and lateral-to-medial capsular shift. In some patients, like overhead athletes, in whom excessive capsular tensioning and plication should be avoided, the suture coming from the anchor should be passed only around the labrum at the same location as the anchor placement (Fig. 4).

**Fig. 5:**
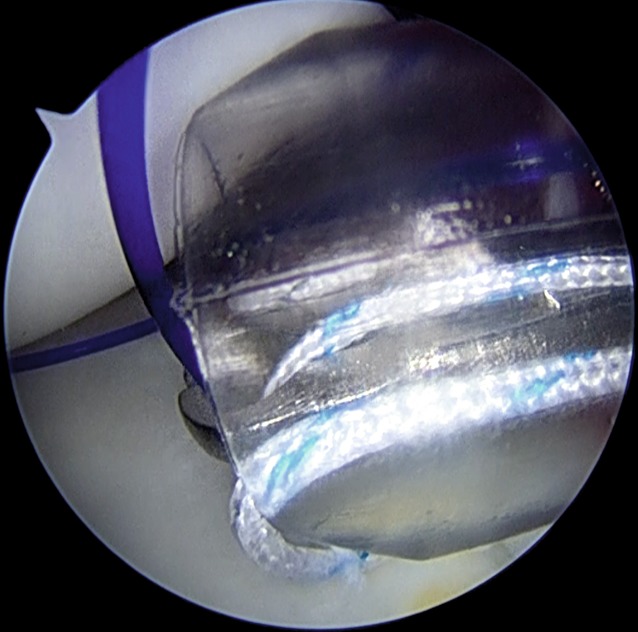
Arthroscopic view of the suture shuttle, exiting through the hook, and the suture from the anchor grasped together through the cannula, to avoid entanglement

In some cases, the labrum may be intact and the pathology may be related only to a capsular detensioning. In these cases, the capsular tissue may be lightly abraded with a rasp or with a shaver and capsular retensioning is carried out using the labrum like an anchor. Specifically, we use a spectrum hook to pass through the capsule and labrum or through the labrum only, depending on the type of lesion and on the patient features; once the hook is passed, the shuttling suture (PDS) is released. The shuttling suture and the one suture from the anchor are then retrieved at the same time to avoid any tangling (Fig. 5).

**Fig. 6:**
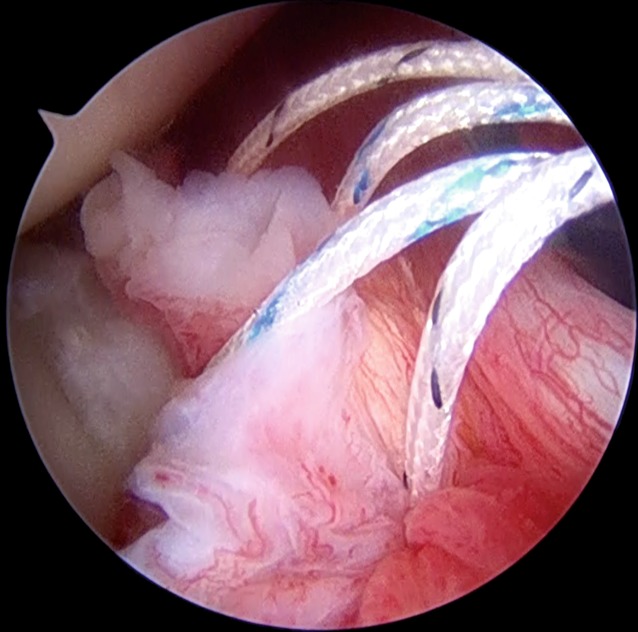
The sutures are all passed through the tissue in a simple configuration. In this case the MIBA stitch (one mattress and one simple suture) is shown

Subsequently, the permanent suture is passed through the tissue and both sutures from the anchor are inside the posterior cannula. The suture that has been passed through the soft tissue is used as the post limb. The sutures are then tied; we prefer to place the knot away from the glenohumeral joint. If we use a double-loaded anchor, all the passages are repeated for the second suture. In the case of a very stretched, loosened tissue, a reinforced configuration of sutures may be used, e.g., the MIBA stitch ([7]; Fig. 6).

**Fig. 7:**
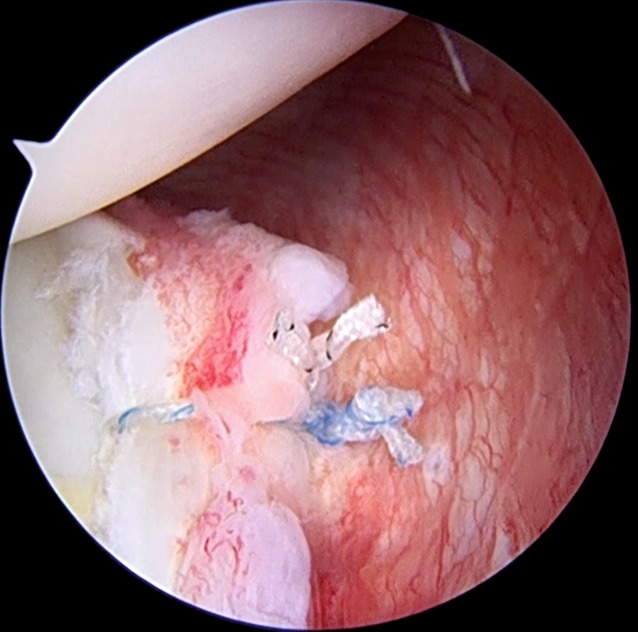
Final repair. The posterior labrum and posterior capsule are repaired to the glenoid rim

When the repair is finished (Fig. 7), we close the posterior portal to prevent stress-riser formation within the posterior capsule. To perform this, the posterior cannula is withdrawn just outside the posterior capsule, a 45° suture hook loaded with a PDS suture is used to penetrate one side of the portal incision, and the suture is retrieved from the opposite side with a penetrating grasper; the sutures are then tied inside the cannula, but outside the posterior capsule.

### Further techniques

We do not suggest routinely making a rotator interval closure at the end of the procedure; however, the surgeon should be able to treat pathologies that may be associated with a posterior instability, particularly multidirectional instability or anterosuperior labral tear [28].

If a capsular tear is associated with a humeral avulsion (PHAGL) it must be repaired with a technique that it similar to the “remplissage technique”: One or two suture anchors are placed in the previously abraded humeral head in the normal capsular insertion and the sutures are passed through the torn capsule to reinsert it to the bone and restore the proper tension of the posteroinferior capsular ligamentous system. In the case of a longitudinal tear, a side-to-side suture can be performed. Typically the knots are closed outside the capsule.

Associated bony lesions of the humeral head should also be evaluated. Patients with posterior shoulder instability could have an osteochondral defect involving the anterior aspect of the humeral head (McLaughlin or reverse Hill–Sachs lesion).

It has been reported that lesions involving less than 20% of the articular surface did well, for the most part, with nonoperative treatment [10]. However, reverse Hill–Sachs lesions tend to involve more of the articular surface compared with their posterior counterparts. Therefore, some authors hold that lesions involving as little as 10% of the articular surface may be clinically significant and require direct intervention [25]. In cases in which this defect is between 10 and 30% of the articular surface, an arthroscopic soft tissue procedure associated with posterior labral and capsular repair has been proposed. In particular, some authors reported a technique suturing the middle glenohumeral ligament (MGHL) [9], while others advocate a technique suturing the distal part of the subscapularis tendon [18] into the defect. The pearls of this surgical technique consist in using a 70° scope through the posterior portal, and suturing the MGHL or the subscapularis tendon into the humeral head defect working from the anterosuperior portal. A burr or a curette is used to abrade the humeral head defect avoiding being too aggressive. Suture anchors (one or two according to the size of the defect) are inserted into the humeral head defect. All the sutures should be passed in a mattress fashion through the soft tissue before tying knots.

## Postoperative management

Postoperative rehabilitation is crucial for maximizing clinical and functional outcome. The operated arm is immobilized in a sling with 15° of external rotation for 6 weeks. For the first 3 weeks, the patients remove the sling three to four times a day to move actively the elbow, wrist, and fingers. After 3 weeks, patients start passive mobilization for shoulder elevation in the scapular plane and shoulder external rotation. Internal rotation recovery is initiated 6 weeks after surgery, when patients can start active range of motion and progressive strengthening of the rotator cuff, deltoid, and scapular stabilizers.

## Discussion

Posterior shoulder instability is a relatively rare condition. The diagnosis is difficult because patient history and clinical examination are not straightforward. Very often, patients present for observation because of a painful shoulder, dysfunction, and rarely because of symptomatic instability. MRA may be used to confirm the diagnosis, but in some situations the findings can be negative. Shoulder arthroscopy gives us the possibility to identify and treat the soft tissue lesions responsible for this type of instability with a good rate of success. To date, there are few studies reporting the results of isolated arthroscopic posterior stabilization. Furthermore, it is difficult to analyze the results because the surgical techniques used are different (capsular plication, repair of labrum with or without anchor, repair of labrum associated with capsular plication) as are the instabilities (posterior, posteroinferior, multidirectional with a posterior predominance). In 2003, Kim et al. [16] reported on a series of patients treated because of unidirectional recurrent posterior instability. After surgery, all the patients were able to resume sports activities. One case of failure because of recurrent instability was reported. Radkowski et al. [26] compared throwers and non-throwers in a group of athletes with unidirectional posterior shoulder instability who underwent arthroscopic capsulolabral repair or plication with or without suture anchors. A 10% failure rate was reported, and no differences were found between the two groups of athletes.

Looking at the studies collectively, we see that a success rate of more than 90% is reported with a large portion of patients able to resume work and sports activities. Failures are related to recurrence of pain or instability. Recurrent instability has been reported in a range of 6–10% of patients [2, 3, 5, 19, 23, 24]. A 20% rate of recurrence was previously reported using multiple suture technique with monofilament stitches [20]. The persistence of pain after surgery is another sign of failed surgery. This has been reported on average in 6% of patients [2, 3, 5, 25]. Pain may be related to unaddressed associated lesions, such as osteochondral defect or partial rotator cuff tear. However, the presence of a posterior chondral glenoid defect in particular seems to be a predictor of poor outcome. The prevalence of concomitant chondral damage associated with posterior instability is variable and seems to increase with the age of the patients [3]. If this lesion is found during arthroscopic repair, the labrum is advanced into the cartilage defect to make the defect extra-articular. However, in such cases the outcomes are still worse than in cases with posterior labral tears and no significant cartilage defect. Furthermore, in cases of bipolar (humeral and glenoid) cartilage defect some authors suggest not performing posterior labral repair because bad results are to be expected [3].

## Practical conclusion

- Posterior shoulder instability is a rare clinical condition that encompasses different pathologies involving the labrum, the capsule, bony lesions, and locked posterior dislocation.
- Diagnosing posterior instability may be difficult because patients often report vague symptoms not linked to a clear history of traumatic shoulder dislocation.
- Physical examination comprising clinical tests (i. e., jerk test, Kim test, and reinterpreted O’Brien test) and imaging (i. e., magnetic resonance arthrography) can facilitate the diagnosis.
- Arthroscopy can be used for a complete evaluation of the joint and allows for the treatment of soft tissue lesions.
- Poor outcomes after soft tissue repair have been reported in cases of concomitant posterior glenoid chondral defects.
